# Rictor mediates p53 deactivation to facilitate the malignant transformation of hepatocytes and promote hepatocarcinogenesis

**DOI:** 10.1186/s12967-023-04799-9

**Published:** 2023-12-18

**Authors:** Chun Wang, Hui Kang, Yun Yi, Yang Ding, Fan Wang, Jie Luo, Mingliang Ye, Yinghui Hong, Chao Xia, Junwei Yan, Lan Liu, Jing Liu, Zibiao Zhong, Zhonglin Zhang, Qiu Zhao, Ying Chang

**Affiliations:** 1 Department of Gastroenterology, Zhongnan Hospital of Wuhan University, Wuhan, 430071 China; 2 Hubei Clinical Center and Key Laboratory of Intestinal and Colorectal Diseases, Wuhan, 430071 China; 3 Department of Geriatrics, Xinhua Hospital, Shanghai Jiao Tong University School of Medicine, Shanghai, 200092 China; 4 Institute of Liver Diseases, Tongji Hospital, Tongji Medical College, Huazhong University of Science and Technology, Wuhan, 430030 China; 5 Department of Gastroenterology, Wuhan Central Hospital, Tongji Medical College, Huazhong University of Science and Technology, Wuhan, 430030 China; 6 Transplant Center of Wuhan University, Institute of Hepatobiliary Diseases of Wuhan University, Zhongnan Hospital of Wuhan University, Wuhan, 430071 China; 7 Department of Hepatobiliary and Pancreatic Surgery, Zhongnan Hospital of Wuhan University, Wuhan, 430071 China

**Keywords:** Hepatocellular carcinoma, Rictor, *TP53*, microRNA-192, Nucleocytoplasmic shuttle, Wild-type, Mutant

## Abstract

**Background:**

Mutations in *TP53* gene is considered a main driver of hepatocellular carcinoma (HCC). While *TP53* mutations are the leading cause of p53 dysfunction, their occurrence rates may drop to approximately 10% in cohorts without hepatitis B virus and aflatoxin exposure. This observation suggests that the deactivation of wild-type p53 (p53^wt^) may be a critical factor in the majority of HCC cases. However, the mechanism undermining p53^wt^ activity in the liver remains unclear.

**Methods:**

Microarray analysis and luciferase assay were utilized to confirm target associations. Gain- and/or loss-of-function methods were employed to assess alterations in signaling pathways. Protein interactions were analyzed by molecular immunological methods and further visualized by confocal microscopy. Bioinformatic analysis was performed to analyze clinical significance. Tumor xenograft nude mice were used to validate the findings in vivo.

**Results:**

Our study highlights the oncogenic role of Rictor, a key component of the mammalian target of rapamycin complex 2 (mTORC2), in hepatocytes. Rictor exerts its oncogenic function by binding to p53^wt^ and subsequently blocking p53^wt^ activity based on p53 status, requiring the involvement of mTOR. Moreover, we observed a dynamic nucleocytoplasmic distribution pattern of Rictor, characterized by its translocation from the nucleus (in precancerous lesions) to the cytoplasm (in HCCs) during malignant transformation. Notably, Rictor is directly targeted by the liver-enriched microRNA miR-192, and the disruption of the miR-192-Rictor-p53-miR-192 signaling axis was consistently observed in both human and rat HCC models. Clinical analysis associated lower miR-192/higher Rictor with shorter overall survival and more advanced clinical stages (*P* < 0.05). In mice, xenograft tumors overexpressing miR-192 exhibited lower Rictor expression levels, leading to higher p53 activity, and these tumors displayed slower growth compared to untreated HCC cells.

**Conclusions:**

Rictor dynamically shuttles between the nucleus and cytoplasm during HCC development. Its pivotal oncogenic role involves binding and inhibiting p53^wt^ activity within the nucleus in early hepatocarcinogenesis. Targeting Rictor presents a promising strategy for HCC based on p53 status.

**Supplementary Information:**

The online version contains supplementary material available at 10.1186/s12967-023-04799-9.

## Introduction

Primary liver cancer ranked as the third leading cause of death from cancer worldwide in 2020 [1, 2]. Hepatocellular carcinoma (HCC) is the predominant form of liver cancer and accounts for 75–85% of cases. Approximately 70% of HCC patients are ineligible for curative treatments, such as liver resection due to late diagnosis at advanced stages [3, 4]. The combination of the immune checkpoint inhibitor (anti-programmed death ligand-1 antibody) atezolizumab and the anti-angiogenic agent (anti-vascular endothelial growth factor antibody) bevacizumab has been accepted as the first-line agent and showed superiority over sorafenib and lenvatinib in individuals with unresectable HCC [4–7], yet overall survival at 12 months was only 67.2% and median progression-free survival was 6.8 months, which is far from satisfactory [5]. A deeper understanding of the molecular mechanisms underlying this disease is needed for the development of novel therapeutics.

HCC arises from a combination of genetic alterations and epigenetic modifications. *TP53* stands out as one of the few essential driver genes in HCC and also the most frequently mutated gene in human cancer [8]. Initially identified in 1979 [9], p53 received unprecedented attention and was called ‘the guardian of the genome’ due to its remarkable antitumor effects, which cover almost all aspects of cancer hallmark regulation [10]. *TP53* mutation rates can reach 50% in HCC cohorts with hepatitis B virus (HBV) and aflatoxin exposure [11–13], while this percentage may decline to 10% or even lower in cohorts without exposure to such mutagens. Although p53 is uniformly mutated in 28.49% HCC according to the TCGA database, a majority of HCC cases still retain the wild-type *TP53* gene. Moreover, although *TP53* mutations are commonly considered late events in advanced and larger HCCs [8], a significant upregulation of p53 is observed in adjacent non-neoplastic tissues with liver cirrhosis during the early stages of HCC development [14, 15]. It is worth noting that the prevalence of positive p53 expression exceeds the *TP53* mutation rates in HCCs [12, 14]. The elevation in p53 expression cannot be solely attributed to *TP53* mutations, even though *TP53* mutations result in prolonged p53 half-life and the accumulation of mutant p53. This phenomenon raises an intriguing paradox: even though many HCC patients retain the wild-type p53 (p53^wt^), p53 fails to effectively impede the transition from precancerous to cancerous stages. Apart from the possibility that a single gene may be insufficient to determine HCC occurrence, another plausible explanation is that the loss of functional p53 is achieved by p53^wt^ inactivation rather than *TP53* mutation, suggesting the presence of potent p53 inhibitors during the progression from precancerous to cancerous stages in HCCs.

Targeting p53 has long been challenging due to the lack of typical drug target characteristics [10, 16]. Identifying negative regulators of p53 and developing strategies to shield p53 from its inhibitory effects provides alternative routes to restore p53 functions [16]. MDM2 is widely recognized as the most well-validated p53 inhibitor. However, targeting MDM2 presented certain difficulties particularly in the context of HCC, as the liver tissue appears to be relatively insensitive to alterations in MDM2—liver tissue remains phenotypically normal after MDM2 ablation, while colon and bone marrow tissues undergo potent apoptosis, suggesting that the MDM2-dependent deactivation of p53^wt^ may vary across organs [17, 18]. Thus, the liver may possess relatively tissue-specific mechanism to inhibit p53 activity during hepatocarcinogenesis. Given the considerable global prevalence of HCC, treatment regimens that are effective in only a small subset of HCC with dysfunctional p53 are still expected to benefit a large number of patients.

Here, we provide substantial evidence that Rictor, a well-known component of the mammalian target of rapamycin complex 2 (mTORC2), has significant oncogenic properties in HCC development through p53^wt^ deactivation. Our study reveals the oncogenic role of the miR-192-Rictor-p53-miR-192 axis, constituting a positive feedback signaling pathway, in hepatocarcinogenesis. The liver-enriched miR-192 emerges as a strategic candidate for Rictor targeting. Through investigating the p53 inactivation during the transformation of normal hepatocytes into hepatoma cells, our aim was to provide insights for liver cancer prevention and pre-HCC treatment strategies in patients with chronic liver diseases. Furthermore, our findings first unveil the dynamic subcellular distribution of Rictor, which shuttles between the cytoplasm and nucleus at various stages of hepatocarcinogenesis, thereby determining its distinct pivotal functions in driving HCC progression. This discovery opens new strategies for personalized HCC treatments tailored to the specific disease stages.

## Materials and methods

### Patient tissues and cell culture

All materials used in this study were obtained under an approved Institutional Review Board protocol. Informed consent was obtained from all human subjects. American HCC samples and their adjacent background liver (BL) tissues were collected as described previously [19], and six additional pairs were newly included (n = 26). Surgically verified Chinese HCC tissues and BL tissues were obtained from the Departments of Surgery of Tongji Hospital of Huazhong University and Zhongnan Hospital of Wuhan University (n = 32). All collected samples were used for miR-192 detection. For Rictor protein detection, 12 pairs each of the American and Chinese samples were randomly selected. An additional 10 fresh HCC samples for nuclear and cytoplasmic fractionation experiments were collected from the Department of Surgery of Zhongnan Hospital of Wuhan University. Hepatoma cell lines Huh7, Hep3B and HepG2 were purchased from the China Center for Type Culture Collection (CCTCC) of Wuhan University, and the PLC/PRF/5, BxPC-3 and HeLa cell lines were purchased from the American Type Culture Collection (ATCC). Cells were cultured as previously described [20]. Huh7 cells were selected with 400 µg/ml G418 (Biofeng Lab. China, A110-02) for three weeks to obtain Huh7 cells stably transfected with pCMV-p53.

### MiRNA microarray analysis

The expression profiles containing 723 human miRNAs were determined in seven hepatoma cell lines and three cases of normal primary human hepatocytes (PHHC) using human miRNA array (Agilent, Santa Clara, CA,USA) as previously described (http://www.ncbi.nlm.nih.gov/geo/, Gene Expression Omnibus public database, accession number, GSE20077) [20]. The raw intensity data were further analyzed using GeneSpring10.0 software (Agilent).

### Luciferase assay

Sixty-bp fragments of the WT or a mutant Rictor 3’UTR sequences (Additional file 2: Table S1) were cloned into the pMIR-REPORT luciferase plasmid (Ambion, Thermo Fisher Scientific, USA). Luciferase assays were performed as previously described [20].

### Reverse transcription and quantitative real-time polymerase chain reaction

MiRNA expression levels were determined by reverse transcription and TaqMan quantitative real-time polymerase chain reaction (qPCR) as previously described [20]. qPCR for mRNA detection was performed by using UltraSYBR Mixture (CWbio, Beijing, China) according to the instructions. RNU6B was used for miRNA qPCR normalization. All data were analyzed by the 2^−ΔΔCt^ method. The primer sequences for qPCR are listed in Additional file 2: Table S2.

### MiRNA-related reagents, siRNAs, plasmids and transfection

The reagent information is listed in Additional file 2: Table S3. Cell transfections were performed with Lipofectamine 2000 according to the manufacturer’s instructions. All tests were performed 48 h after transfection unless specified otherwise.

### Antibodies, western blotting and coimmunoprecipitation

The antibody information is listed in Additional file 2: Table S3. The methods of protein preparation and coimmunoprecipitation (co-IP) are included in Additional file 3: Materials. Western blotting was performed as described previously [20].

### Fluorescence microscopy and confocal microscopy

The methods are included in the Additional file 3: Materials.

### Immunohistochemical staining and quantification

The methods are included in the Additional file 3: Materials.

### Sanger sequencing

The methods are included in the Additional file 3: Materials.

### Animal experiments

Wistar rats (male, 7–8 weeks old, 200–220 g) and BALB/c nude mice (male, 3–4 weeks old, 10–12 g) were purchased from Beijing Vital River Laboratory Animal Technology Co., Ltd. The animal protocols were approved by the Institutional Animal Care and Use Committee (IACUC) of Wuhan University. All animal experiments were carried out according to the guidelines of the Center for Animal Experiment/Animal Biosafety Level-III Laboratory. Chol-miR-192 or Chol-miR-NC were purchased from RiboBio Co., Ltd, Guangzhou, China. The methods for establishing fibrosis, cirrhosis and HCC rat models are illustrated in Additional file 1: Figure S1.

### Bioinformatic analysis

The downloaded cohort data were for 376 liver HCC (LIHC) patients whose clinical data are available from The Cancer Genome Atlas (TCGA) (https://portal.gdc.cancer.gov/) (Additional file 2: Table S4). The method of bioinformatic analysis is included in Additional file 3: Materials.

### Statistical analysis

Quantitative data are presented as the mean ± SD and were analyzed by Student’s *t*-test or one-way ANOVA. Qualitative data are representative of three experiments. *P* < 0.05 was considered to indicate statistical significance.

## Results

### Rictor exhibits oncogenic effects depend on p53 status in vitro

**Fig. 1:**
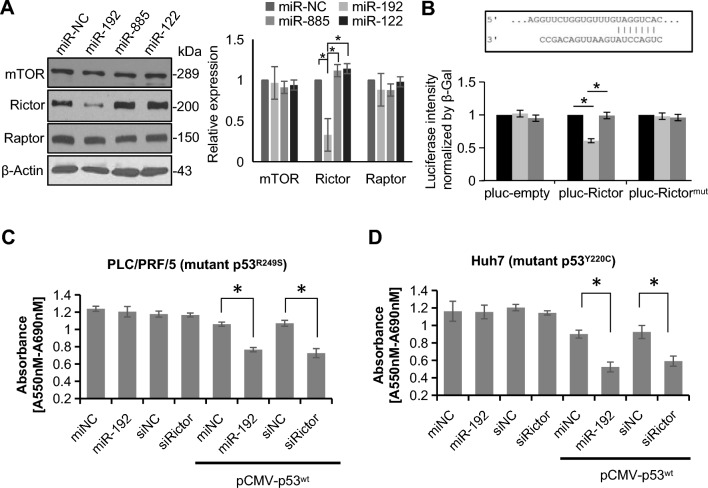
Rictor exhibits a p53^wt^-dependent HCC-promoting effect in vitro. **A** Western blotting assay and protein quantitative analysis in Huh7 cells: effect of pre-miR-192 on mTOR, Rictor, and Raptor levels. Pre-miR-NC was the negative control. Two unrelated microRNAs, pre-miR-885 and pre-miR-122, were used as parallel controls. **B** TargetScan-predicted miR-192 binding site at Rictor 3’UTR. Luciferase reporter assay showed that miR-192 significantly inhibited miR-192 on the reporter vectors but not in other vectors. Luciferase activity was standardized to that of β-Gal as a control. **C, D** Cell proliferation assessed by MTT assay 72 h after transfection with miR-192 precursors or siRNAs for Rictor (siRictor) in PLC/PRF/5 and Huh7 cells. MiR-NC and siNC were set as negative controls (NC). *P < 0.05; ns, not significant

Our prior investigation highlighted the pivotal role of liver-enriched miR-192 in autophagy within hepatoma cells [21]. Based on this discovery, we explored the potential correlation between miR-192 and key molecules involved in the autophagy-related mTOR signaling pathway (mTOR, Raptor, and Rictor). Rictor was significantly suppressed by miR-192 at both the mRNA and protein levels (Fig. 1A). We further confirmed that Rictor is a direct downstream target of miR-192 by a luciferase reporter assay (Fig. 1B), confirming our initial prediction made by bioinformatic prediction (Additional file 1: Fig. S2B) [22].

MiR-192 has been reported to play an oncogenic role in a p53 status-dependent manner [23]. We hypothesized that Rictor mediates the interaction between miR-192 and p53^wt^. Silencing Rictor alone had a relatively modest or negligible impact on cell growth of *TP53*-mutated hepatoma cell lines, including PLC/PRF/5 (with a *TP53* p.R249S mutation) and Huh7 (with a *TP53* p.Y220C mutation) (Fig. 1C, D). However, when p53^wt^ was restored by co-transfecting *TP53*-mutated hepatoma cells with pCMV-p53^wt^, Rictor deprivation markedly hindered cell growth (Fig. 1C, D). Similar effects were observed in the pancreatic cancer cell line BxPC-3 (with a *TP53* p.Y220C mutation) (Additional file 1: Fig. S3A). In addition, Rictor was predominantly expressed in Huh7 cells among the cell lines tested (Hep3B with p53-null; HepG2 with p53^wt^, and Huh7 with p53^Y220C^) (Additional file 1: Fig. S3B), consistent with previous reports of chromosomal gain of *RICTOR* in Huh7 cells [23]. In HepG2 cells that possess p53^wt^ but express only trace amounts of endogenous Rictor (Additional file 1: Fig. S3B), restoring miR-192 or silencing Rictor showed detectable but not significant inhibition of cell growth (Additional file 1: Fig. S3C). These findings suggest that Rictor exerts its oncogenic effects by attenuating the antitumor activity of p53^wt^, which at least partially explains why miR-192 exhibits p53^wt^-dependent antitumor effects [24].

### Rictor exhibits mTOR-dependent nuclear mutual binding with p53^wt^ and suppresses p53^wt^ activity

**Fig. 2:**
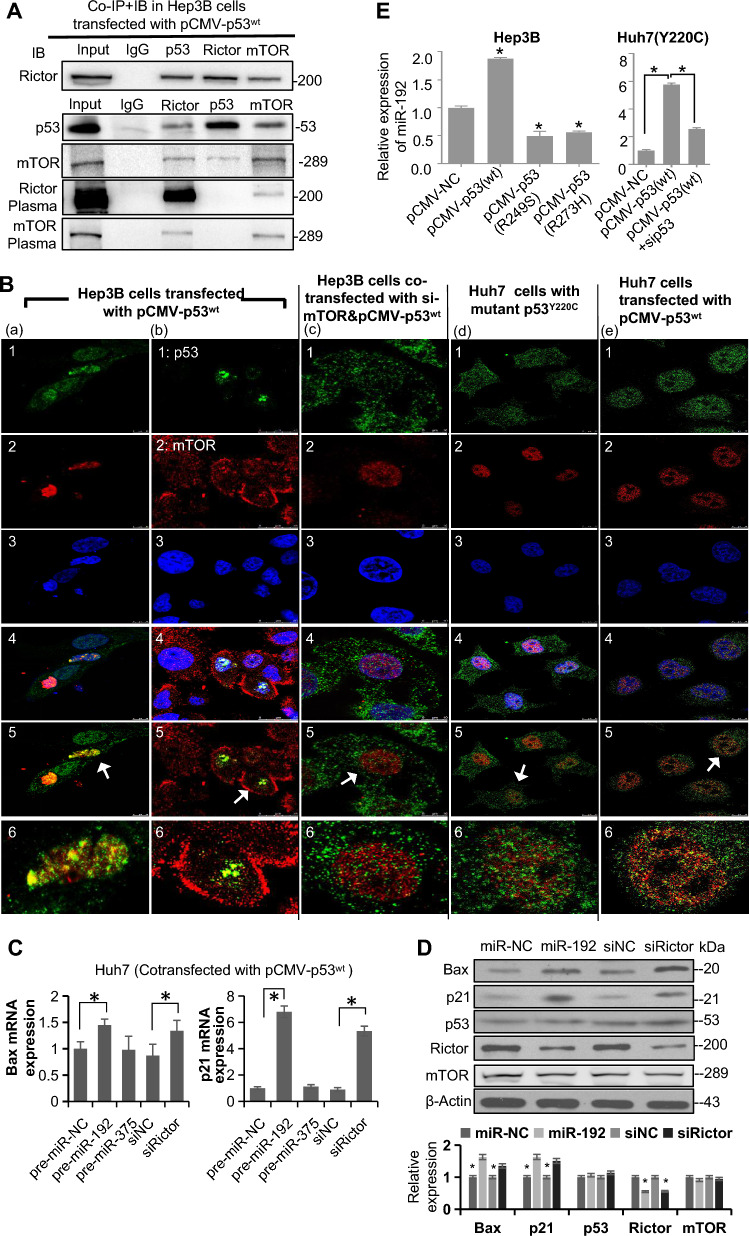
Rictor forms a mutual binding interaction with wild-type p53. **A** Coimmunoprecipitation (Co-IP) and immunoblotting (IB) in Hep3B cells 48 h after transfection with pCMV-p53^wt^. **B** Confocal microscopy (CM) images of Rictor, p53, and mTOR in HCC cell lines. 1: Green Rictor staining (except column (b), showing green p53 staining); 2: Red p53 staining (except column (b), showing red mTOR staining); 3: Blue Hoechst nuclear staining; 4: Merged staining of 1 + 2 + 3; 5: Merged staining of 1 + 2; 6: Magnified regions are indicated by white arrows. **C** RT-qPCR analysis of Bax and p21 mRNA expression in Huh7 cells after 24 h of transfection with pCMV-p53^wt^. An irrelevant miRNA pre-miR-375 served as a parallel control. **D** Western blotting assay and densitometric analysis in Huh7 cells (transfected with pCMV-p53^wt^) 48 h after transfection. **E** RT-qPCR measurement of miR-192 expression levels in HCC cells 48 h after transfection. *P < 0.05; ns, not significant

We tested the hypothesis that Rictor interacts with p53^wt^ through mutual binding by performing a coimmunoprecipitation (co-IP) assay. Using the respective antibodies, we successfully co-precipitate Rictor and p53^wt^ in Hep3B cells (*TP53*-null, p53−/−) transfected with pCMV-p53^wt^ (Fig. 2A). Additionally, fractionation studies showed that the Rictor antibody was unable to precipitate p53^wt^ when specifically analyzing cytoplasmic proteins, suggesting that the interaction between p53^wt^ and Rictor primarily occurs in the nucleus (Fig. 2A). This phenomenon was further confirmed by confocal microscopy (CM). In pCMV-p53^wt^-transfected Hep3B cells, Rictor was distributed in both the cytoplasm and the nucleus, whereas p53 was mainly located in the nucleus (Fig. 2Ba); Rictor colocalized well with p53^wt^ in the nucleus, as shown by the merged yellow staining on the blue background. The co-IP assay and CM consistently demonstrated that Rictor interacts with p53^wt^ in the nucleus through mutual binding. Notably, Huh7 cells harboring only mutant *TP53*^Y220C^ exhibited dispersed distributions of Rictor and p53^Y220C^ in the nucleus (Fig. 2Bd). However, when Huh7 cells were stably transfected with pCMV-p53^wt^, leading to the coexistence of p53^wt^ and p53^Y220C^, colocalization between Rictor and p53 could be observed again, albeit only partially (Fig. 2Be). These findings indicate that Rictor selectively binds p53^wt^ but not mutant p53^Y220C^ in the nucleus. *TP53* has several common mutant forms, such as R248Q, Y220C, R249S, and R175H; therefore, further structural studies are warranted to decipher the precise nature of the association between p53 and Rictor.

Given that Rictor is a critical component of mTORC2, we performed an investigation to determine whether mTOR is involved the binding of p53^wt^ and Rictor. The co-IP results showed that mTOR could be pulled down by Rictor and p53 antibodies (Fig. 2A). Additionally, CM confirmed the colocalization of mTOR with p53^wt^ (Fig. 2Bb) and Rictor (Additional file 1: Fig. S4A) in the nucleus. After mTOR deprivation, the colocalization of p53^wt^ and Rictor was markedly impeded (Fig. 2Bc; Additional file 1: Fig. S4B). The above findings indicate that mTOR plays a key role in the interaction between Rictor and p53^wt^.

Furthermore, rescue of miR-192 or silencing of endogenous Rictor induced upregulation of Bax and p21 (two p53-regulated downstream molecules) at mRNA and protein levels in pCMV-p53^wt^-transfected Huh7 cells (Fig. 2C, D). The levels of p53 and mTOR proteins were not significantly changed (Fig. 2D), implying that Rictor inhibited the activity of p53 as a transcription factor through protein–protein interaction without affecting p53 expression levels. Besides, restoring p53^wt^ expression could increase miR-192 levels in Hep3B and Huh7 cells, whereas the overexpression of mutant p53 had a dominant-negative effect on miR-192 levels (Fig. 2E). Thus, a positive feedback loop between miR-192 and p53^wt^ (i.e., miR-192-Rictor-p53-miR-192 axis) was established in HCC. This feedback loop represents a relatively tissue-specific regulatory mechanism and underscores the interplay between Rictor and p53 signaling pathways during HCC carcinogenesis.

### Rictor upregulation throughout hepatocarcinogenesis in vivo

**Fig. 3:**
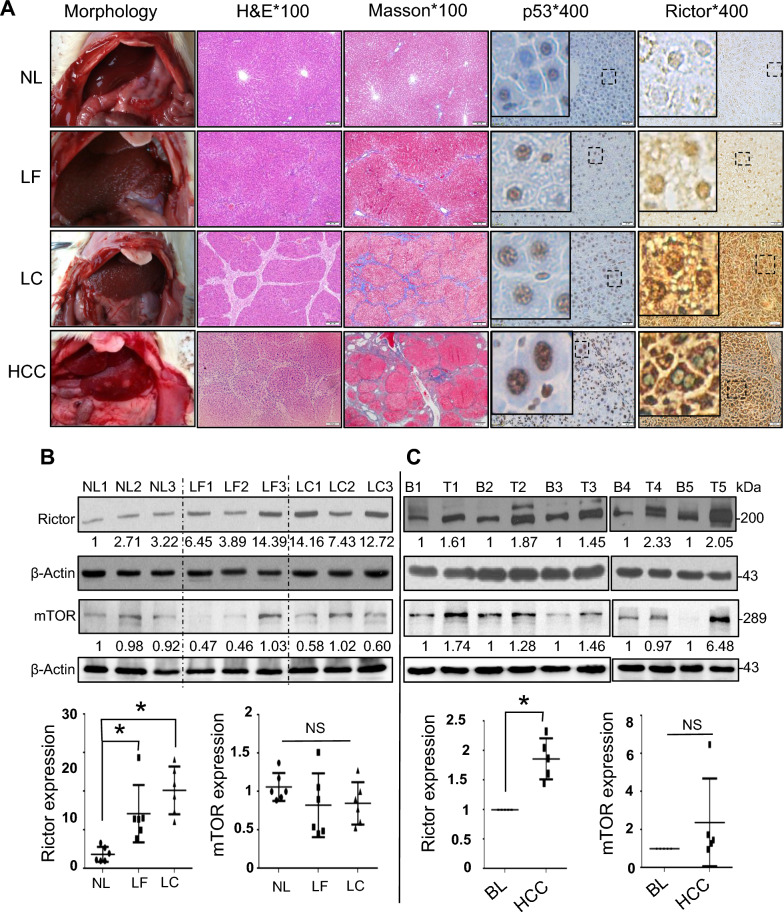
Dysregulation of the miR-192-Rictor-p53-miR-192 axis during hepatocarcinogenesis. **A** Morphology, H&E staining, Masson staining, and immunohistochemical staining changes of p53 and Rictor in liver tissues of rat cancer models at different stages: normal liver (NL), liver fibrosis (LF), liver cirrhosis (LC), and hepatocellular carcinoma (HCC). **B, C** Comparison of protein expression profiles of Rictor and mTOR in liver tissues of rat models (**B**) and clinical patients (**C**) at different stages of hepatocarcinogenesis by western blotting and quantified via densitometric analysis (n = 6 for NL, LF, and LC each; three representatives were shown in each group; n = 5 for HCCs and background liver (BL) tissues). H&E: magnification × 100. IHC staining: magnification × 400. *P < 0.05; **P < 0.01; *ns* not significant

To explore dynamic changes in Rictor and p53^wt^ during hepatocarcinogenesis, we established rat models to simulate the pathological development of HCC—from normal liver to fibrosis/cirrhosis and then to liver cancer (Fig. 3A). We found that Rictor levels gradually increased in the early stages of HCC, as indicated by the presence of Rictor levels in precancerous tissues (fibrosis and cirrhosis) vs. in normal tissues (Fig. 3A, B). Our results indicate that Rictor upregulation is an early event and is consistently observed during hepatocarcinogenesis in HCC. MiR-192 showed synchronous downregulation, exhibiting an opposite trend to Rictor (Fig. S5). As for p53, we found that it gradually increased during liver tumorigenesis and was mainly localized in the nuclei of all HCC cells (n = 5) (Fig. 3A). Sequencing analysis of the *TP53* gene indicated that the *TP53* coding sequences in all five rat HCCs were wild type (Additional file 1: Fig. S6).

In contrast, mTOR expression did not show a consistent overall increasing trend throughout the progression from normal liver (NL) to liver fibrosis (LF) and cirrhosis (LC) tissues. In rat models, although higher mTOR expression was generally observed in LC vs. LF tissues (4/5), this difference did not achieve statistical significance (*P* = 0.215) (Fig. 3B). In clinical patients, a significant increase in Rictor levels was observed in HCC vs. background liver (BL) tissues; however, the increase in mTOR expression did not reach statistical significance (Fig. 3C). Although Rictor and mTOR may function as a complex, the upregulation of Rictor appears to be more closely related to p53 activity than mTOR, particularly in precancerous tissues where mTOR levels have not yet significantly increased.

### P53-dependent nucleocytoplasmic trans-localization of Rictor during hepatocarcinogenesis

**Fig. 4:**
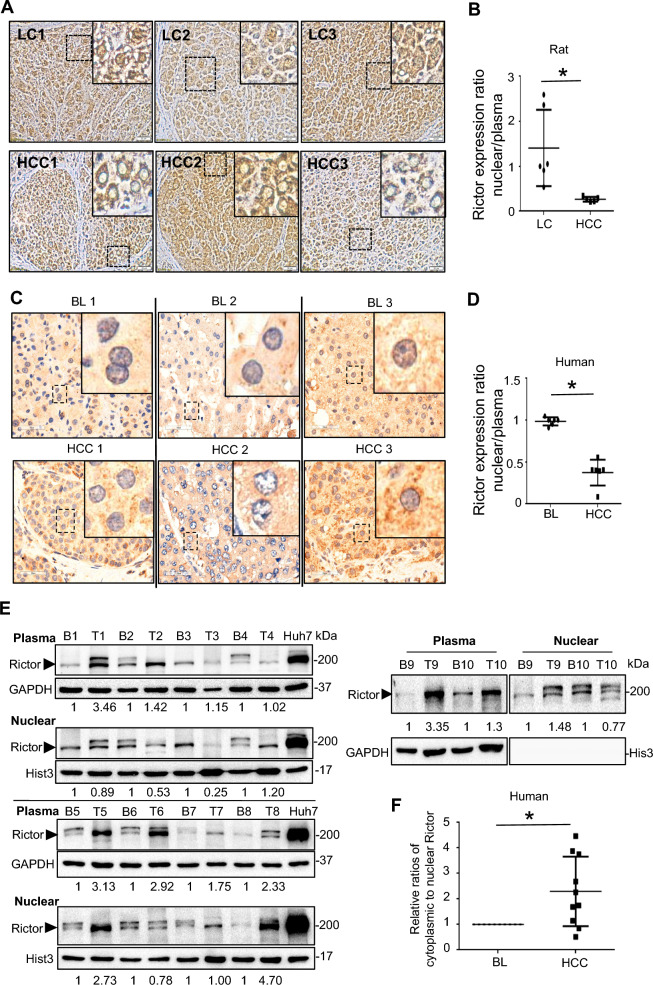
Intracellular translocation of Rictor during hepatocarcinogenesis. **A–D** Immunohistochemical (IHC) staining and corresponding quantitative analysis of Rictor in (**A, B**) three representative rat liver cirrhosis (LC) and hepatocellular carcinoma (HCC) tissues, as well as (**C, D**) three representative human HCCs and their background liver (BL) tissues. Magnified regions of (**A**) and (**C**) are highlighted by dashed boxes. Cytoplasmic-nuclear distributional disparities of Rictor are represented by relative ratios of nuclear H-score to cytoplasmic H-score in (**B**) rat LC (n = 6) and HCC (n = 5) tissues, and in (**D**) human BL (n = 6) and HCCs (n = 6) tissues. **E** Western blotting demonstrated cytoplasmic and nuclear Rictor expression in 10 pairs of fresh human HCCs (T: tumor) and their BL tissues (B: background). **F** Quantification of the blot in (**E**) by densitometric analysis. Ratios of cytoplasmic to nuclear Rictor expression in each BL were normalized to 1 (n = 10). Relative ratios of cytoplasmic to nuclear Rictor expression in the HCCs vs. BL tissues were calculated. IHC staining: magnification × 400. *P < 0.05

In rat models, an interesting observation showed that Rictor was distributed in cytoplasmic and nuclear regions of all premalignant cirrhosis samples. However, in HCC, Rictor was overwhelmingly sequestered in the cytoplasmic region (Fig. 4A). This shift in distribution was evidenced by a marked decrease in the nuclear to cytoplasmic H-scores of Rictor staining in HCCs vs. LC tissues (Fig. 4B). The cytoplasmic distribution of Rictor has a similar preference in human HCCs vs. BL tissues; Rictor staining appeared more intense in the HCC cytoplasm than in the nucleus (Fig. 4C, D). These results indicated that Rictor can translocate between the cytoplasm and nucleus, and its localization correlates with different stages of hepatocarcinogenesis. To validate the nucleocytoplasmic translocation of Rictor, we performed fractionation experiments in human tissues. The relative ratios of cytoplasmic to nuclear Rictor expression were normalized to 1 in each BL tissue. In the majority of HCCs examined, the relative ratios exceeded 1 (7/10), thus confirming the occurrence of Rictor nucleocytoplasmic translocation (Fig. 4E, F).

**Fig. 5:**
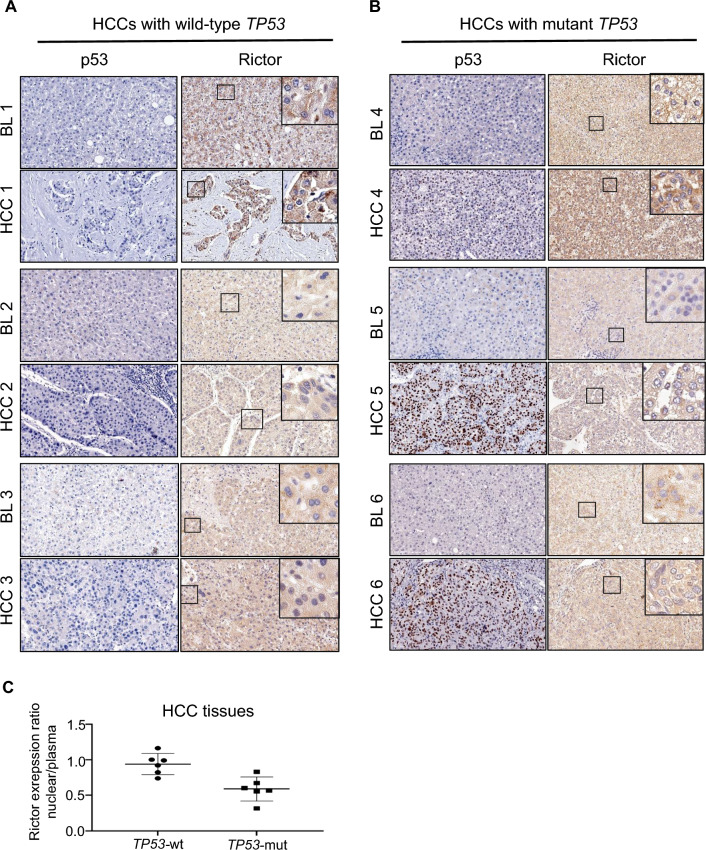
Translocation of Rictor in clinical patients with different *TP53* mutation status. **A, B** Immunohistochemical (IHC) staining of p53 and Rictor in human HCC tissues harboring (**A**) wild-type *TP53* and (**B**) mutant *TP53*. All background liver (BL) tissues exhibited wild-type *TP53*. Magnified regions are indicated by dashed boxes. **C** Differences in the cytoplasmic-nuclear distribution of Rictor are indicated by relative ratios of nuclear H-score to cytoplasmic H-score in HCC tissues with wild-type *TP53* (n = 6) vs. mutant *TP53* (n = 6). IHC staining: magnification × 400. **P < 0.01

Moreover, we noticed a significant accumulation of Rictor, forming granular aggregates in the nuclei of p53^wt^-transfected Huh7 cells by CM observation. In contrast, Rictor was evenly distributed in both the nucleus and cytoplasm of p53^wt^-untransfected Huh7 cells without forming granule-like clusters (Fig. 2Ba). This finding suggests that Rictor colocalizes with p53^wt^ in the nucleus. Similar observations were made in human liver specimens. Rictor was predominantly localized to the nucleus in HCCs with wild-type *TP53* (Fig. 5A), while a pronounced cytoplasmic localization of Rictor was observed in HCCs with mutant *TP53* (Fig. 5B and Additional file 1: Fig. S6). Rictor-stained nuclear to cytoplasmic H-scores were significantly reduced in wild-type *TP53* HCCs vs. mutant *TP53* HCCs (Fig. 5C). All these results suggest that Rictor shuttles from the nucleus (observed in precancerous lesions) to the cytoplasm (observed in HCCs) during malignant transformation, and this translocation may be influenced by the p53 status. Therefore, we proposed a two-stage model to understand the oncogenic effects of Rictor on p53^wt^ HCCs. During precancerous or early cancer stages, p53^wt^ is activated by various cellular stresses, as supported by progressive increase in p53 expression during liver tumorigenesis in rat models. Nuclear Rictor binds to p53^wt^ and inhibits its function, thereby inhibiting cell death and causing malignant transformation of hepatocytes. In late stages when HCC cells have acquired immortality, Rictor is more readily translocated to the cytoplasm and may exhibit p53-independent biological functions.

### Association of universal low miR-192/High Rictor expression with poor overall survival in human HCCs

**Fig. 6:**
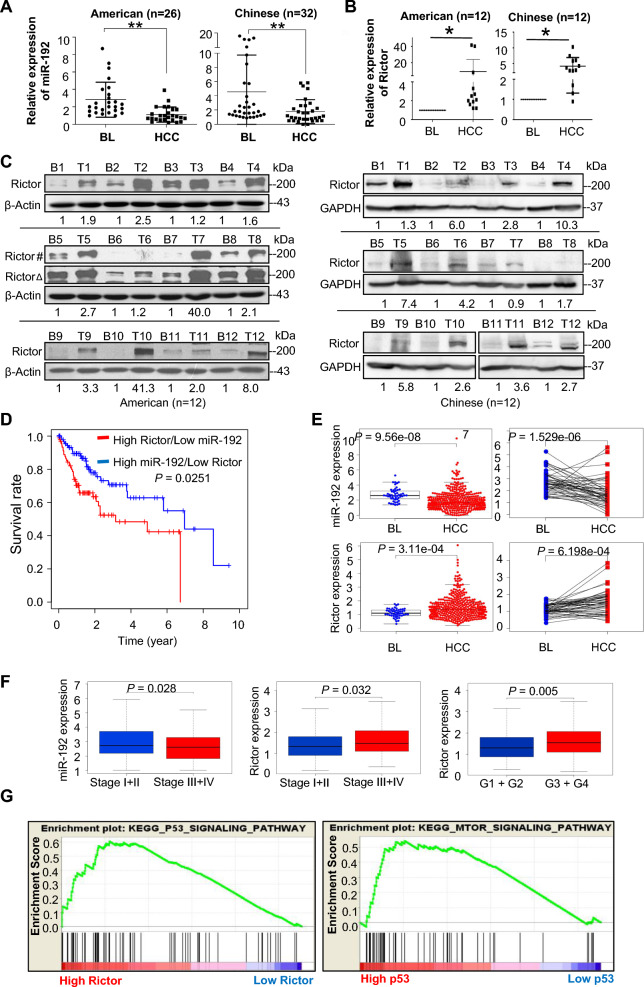
Clinical relevance of the miR-192-Rictor-p53-miR-192 axis in human HCCs. **A** mRNA expression levels of MiR-192 by qPCR in American (n = 26) and Chinese (n = 32) patient cohorts. Comparisons were made between human background liver (BL) tissues and hepatocellular carcinoma (HCC) tissues. **B** mRNA expression levels of Rictor in human HCCs and their corresponding BL tissues by qPCR in the two cohorts (n = 12 each). Photodensity of the BL tissue in each tissue pair was set to 1. **C** Rictor protein expression levels in human HCC (T: tumor) and corresponding background liver (B: background) tissues were measured by western blotting and quantified by densitometric analysis. #, shorter exposure; Δ, longer exposure. **D** Kaplan–Meier curves demonstrate overall survival (OS) between the high Rictor/low miR-192 group and the high miR-192/low Rictor group in HCC patients from The Cancer Genome Atlas (TCGA) database (n = 376). Statistical significance of the correlation between miRNA/mRNA expression and OS was determined by the log-rank test (*P* < 0.05). **E** mRNA expression levels of MiR-192 and Rictor in HCC vs. BL patients based on TCGA data. BL: adjacent background liver. **F** Correlationo of Rictor or miR-192 expression levels with clinicopathologic characteristics, including clinical stage (stage I + II vs. stage III + IV) and histological grade (G1 + G2 vs. G3 + G4). BL: adjacent background liver. **G** Enrichment plots from gene set enrichment analysis (GSEA). All with normalized *P* < 0.01. *P < 0.05; **P < 0.01; *ns* no significant

To verify the clinical significance of Rictor in human hepatocarcinogenesis, we performed analyses using the HCC cohorts in this study. The HCC cohorts were divided into Chinese (with HBV and aflatoxin exposure) and American (without HBV and aflatoxin exposure) groups, considering the distinct genetic backgrounds and HCC etiologies in different populations. MiR-192 consistently exhibited downregulation in HCCs compared with corresponding BL tissues in both cohorts (Fig. 6A). In contrast, Rictor displayed an opposite trend, showing upregulation in the majority of human HCCs (American 12/12 and Chinese 11/12) (Fig. 6B, C). These results indicate that miR-192/Rictor is generally involved in human hepatocarcinogenesis. Notably, miR-192 emerged as the seventh most hepatocyte-enriched miRNAs (Additional file 2: Table S5) and ranked as the fourth most differentially expressed miRNA when comparing liver cancerous hepatocytes (CHs) with normal primary human hepatocytes (PHHCs) in our previous screening (GEO accession number, GSE20077). This finding highlights a liver-specific role of Rictor and miR-192 in the development of hepatocellular carcinoma.

We further analyzed The Cancer Genome Atlas-Liver hepatocellular carcinoma (TCGA-LIHC) data, including 376 HCC patients with available survival status, to examine the clinical relevance of miR-192/Rictor in HCC. Detailed clinical characteristics of these patients are provided in Additional file 2: Table S6. Patients with high miR-192/low Rictor expression levels had significantly longer overall survival (OS) compared with patients with low miR-192/high Rictor expression (Kaplan–Meier method, log-rank *P* = 0.0251) (Fig. 6D). Additionally, this cohort also exhibited upregulation of Rictor and downregulation of miR-192 in HCC vs. adjacent background liver tissues (*P* < 0.001) (Fig. 6E). We performed both univariate and multivariate Cox analyzes using data from the TCGA-LIHC cohort, underscoring that high Rictor expression was an independent risk factor for worse OS (multivariate analysis: hazard ratio = 1.284, 95% confidence interval: 1.023–1.612; *P* = 0.031) (Additional file 2: Table S7, Additional file 1: Fig. S8). Low miR-192 and high Rictor expression were also significantly correlated with advanced clinical stages, wherein higher Rictor expression indicated a worse histological tumor type (Wilcoxon rank sum test: *P* < 0.05) (Fig. 6F). The significance of the association of Rictor expression with clinicopathological characteristics (logistic regression) is also presented in Additional file 2: Table S8.

GSEA analysis confirmed the association between Rictor and p53. Gene sets related to mTOR and p53 signaling pathways were substantially enriched in the high-Rictor expression phenotype; the high-p53 expression phenotype in turn exhibited enrichment of gene sets related to the mTOR signaling pathways (*P-*value < 0.05) (Fig. 6G). Thus, the GSEA results support that Rictor is highly relevant to both the mTOR and p53 signaling pathways. Further details of GSEA comparing the low- and high-Rictor expression datasets are provided in Additional file 2: Table S9.

### MiR-192 inhibits Rictor and the growth of hepatoma xenograft tumors in vivo

**Fig. 7:**
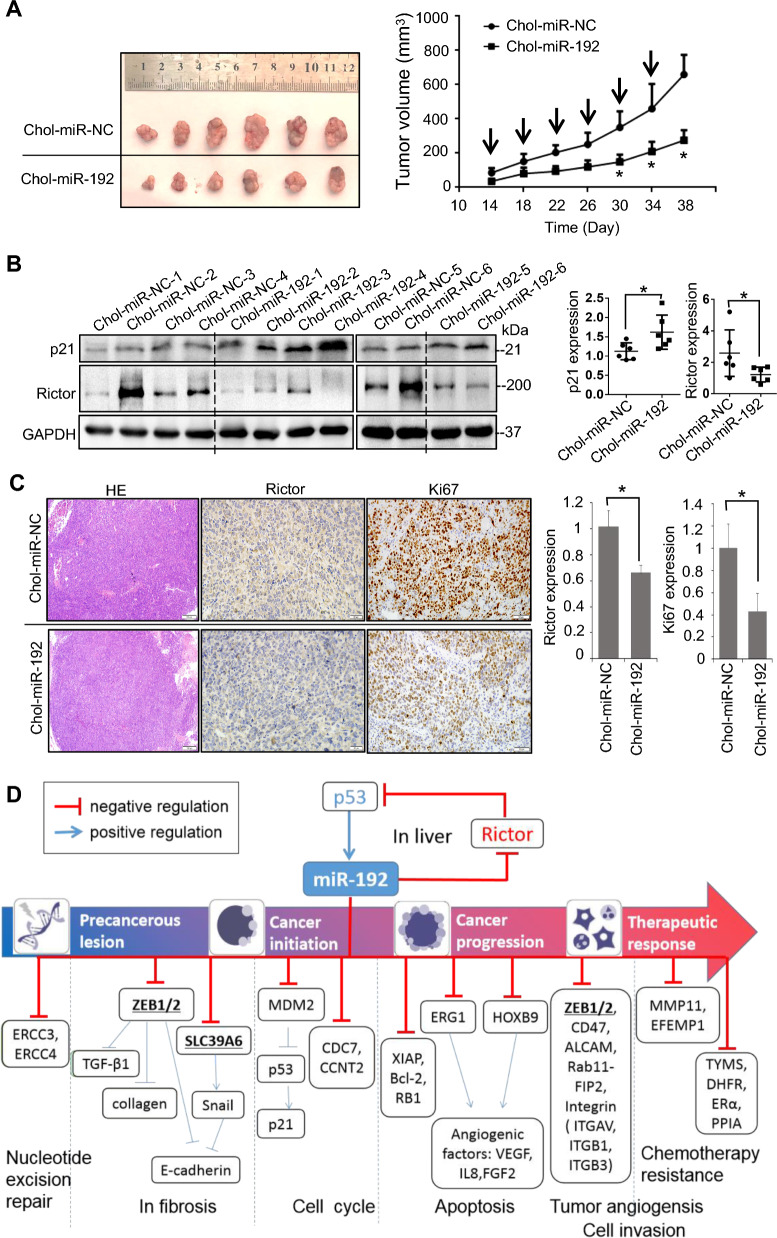
miR-192 suppresses Rictor and inhibits hepatoma xenografts tumor growth in vivo. **A** Morphology of xenografts observed by the In-Vivo Xtreme Imaging System and the tumor growth curves of the indicated groups. Arrows indicate administration of 1 nmol of Chol-miR-192 (n = 6) or negative control (Chol-miR-NC, n = 6). **B** Quantitative analysis of Rictor and p21 expression via western blotting in resected tumors. **C** H&E staining and immunohistochemical staining of Rictor and Ki67 in representative xenograft tissue sections, quantified by densitometric analysis from five randomly selected high-power fields (magnification × 400) per slide (n = 6). **D** Schematic depicting the course of hepatocarcinogenesis and roles of established molecules during the process. *P < 0.05; *ns* no significant

We have previously demonstrated that chemically stabilized miRNAs can efficiently enter hepatoma cells in HCC-loaded nude mice [20]. Subcutaneous hepatoma xenografts were established in the flanks of athymic nude mice using Huh7 cells stably expressing p53^wt^. The intratumoral injection of Chol-miR-192 significantly retarded tumor growth (Fig. 7A). Tumors injected with Chol-miR-192 showed a significant reduction in Rictor expression (Fig. 7B, C), accompanied by an increase in the expression of p53-downstream molecule p21 (Fig. 7B) and a decrease in the levels of Ki67 (Fig. 7C). These data suggest that miR-192 inhibits Rictor and promotes p53 signaling in vivo, thereby contributing to the delayed growth of tumors. The effects of miR-192 in HCC is summarized in Fig. 7D. References are provided in Additional file 2: Table S11.

## Discussion

The most important finding of this study is the confirmation of Rictor's pivotal role in deactivating p53 in HCC. Recently, there has been a growing interest in the oncogenic role of Rictor. Widely recognized as a subunit of mTORC2, it includes mTOR, GβL, Rictor, and mSin1. mTORC2 plays a crucial role in various biological functions, including the regulation of cellular proliferation, metabolism, autophagy, actin cytoskeleton, and cell spreading [25]. We propose that the interaction between Rictor and p53 is critical in hepatocarcinogenesis and highlight the potential liver-specific oncogenic role of Rictor, supported by the following evidence: First, Villanueva et al. reported chromosomal gains of RICTOR in 25% of HCC patients [23], a ratio higher than in other cancer types, such as neuroendocrine prostate cancer (~ 19%), lung cancer (~ 15%), sarcoma (~ 10%), and stomach adenocarcinoma (~ 6%) as analyzed by Kim et al. [26]. Second, our results confirmed the general involvement of Rictor in HCC in both rat models and human specimens, and illustrated Rictor as a potent oncogenic molecule contributing to p53^wt^ deactivation in hepatocytes. Moreover, we found that Rictor is a direct target of miR-192, a miRNA enriched in normal hepatocytes but substantially suppressed in HCCs. All of this evidence indicates that the liver is sensitive to Rictor’s abnormality, emphasizing its potential as a relatively liver-specific oncogene.

We observed an intriguing nucleocytoplasmic redistribution of Rictor during hepatocarcinogenesis. As a subunit of mTORC2, Rictor’s function is intricately associated with its subcellular localization in the context of different stimuli [27]. In the cytoplasm, mTORC2/Rictor appears to be distributed among various organelles (e.g. plasma membrane, endoplasmic reticulum, ribosome etc.) as well as in the cytosol [27]. Functionally, cytoplasmic mTORC2/Rictor phosphorylates Ser473 of AKT to promote cell survival [28], and it participates in the regulation of cytoskeleton and intercellular adhesion to promote cell invasion and metastasis—two malignant phenotypes associated with typical advanced tumors [29]. By contrast, there are few functional studies on nuclear mTORC2 or Rictor. Our result shows that Rictor can be recruited by nuclear p53^wt^ and functional inactivates p53^wt^ in the nucleus by binding with it. As a transcription factor, p53 displays its essential anti-tumor function by transcriptionally activating numerous tumor suppressor genes that prevent cell growth e.g. by cell cycle arrest or promote cell death e.g. by apoptosis. The binding of nuclear Rictor and p53 effectively impedes the transcriptional activation of p53, thus it offers hepatocytes more opportunities for malignant transformation. Devyani et al. recently reported that nuclear mTORC2 promotes histone H3 lysine 56 acetylation (H3K56Ac) at the promoters of glycolytic genes by negatively regulating the recruitment of SIRT6 to regulate their transcription [30]. Anti-cell arrest, anti-apoptosis, and increased glycolysis are all canonical phenotypic changes correlated to the tumor initiation. Based on this evidence, we infer a stage-specific oncogenic mode for Rictor: in the initiation stage of hepatocarcinogenesis, Rictor is prone to interact with p53 in the nucleus, preventing p53-induced apoptosis, maintaining cell survival, and providing sufficient time for the cell to undergo malignant transformation. Once the transformation is achieved, Rictor relocates to the cytoplasm, where it promotes cell invasion and metastasis for the progression of advanced HCC.

We propose that a promising therapeutic strategy could involve reactivating p53^wt^ by releasing it from Rictor in p53^wt^-expressing HCCs. Drugging p53 is a current challenge due to its lack of typical drug target features [16, 31]. Common therapeutic approaches for tumors retaining p53^wt^ expression include targeting MDM2 to inhibit p53^wt^ degradation [16]. Another approach involves the introduction of exogenous p53^wt^; commercially available products like Gendicine provide recombinant adenovirus encoding human *TP53* (rAd-p53), which is a relatively safe and effective treatment option for HCC [32, 33]. However, there is a lack of available MDM2-p53 axis-targeted drugs for HCC patients [34], possibly attributed to the comparatively lower sensitivity of the liver to MDM2 alterations [17, 18]. Moreover, introducing exogenous p53^wt^ in p53^wt^-expressing HCCs may not fully restore p53 function, as p53^wt^ may lose its function through multiple alternative mechanisms, such as binding to nuclear Rictor. Thus, depleting Rictor from the nucleus could be an effective strategy to restore the p53^wt^ function in liver cancers [17, 18, 35].

Our study also highlights the potential of miR-192, an miRNA with robust physiological expression in the liver [22], as a strategic candidate to target Rictor. The work by Liu et al. has validated the effectiveness of miR-192 in suppressing Rictor in nonalcoholic fatty liver diseases [36]. There are multiple approaches to target cancer-related miRNAs, which expand the potential for developing novel Rictor-inhibiting drugs [37]. Despite ongoing efforts to develop mTOR inhibitors, achieving precise targeting of Rictor or mTORC2 remains challenging due to complex protein–protein interactions [38]. Moreover, clinical trials involving mTOR inhibitors face limitations due to their notable side effect profile owing to the metabolic impact of inhibiting the mTOR pathway [39]. The application of Rictor RNA interference demonstrated relatively low toxicity compared with dual mTORC1/2 inhibition [40]. Encouraging preclinical studies have demonstrated that inhibiting mTORC2 through Rictor RNA interference could reduce cell motility in cultured human breast cancer cell lines [41]. Additionally, a nanoparticle-based delivery system loaded with Rictor siRNA showed efficacy in blocking tumor growth in breast cancer models. Thus, targeting liver-enriched miR-192 provides a feasible approach to mTORC2-selective inhibition in HCCs [40].

On the basis of our research, some emerging issues should be further addressed. Firstly, both the mTOR/Rictor signaling pathway and the p53 signaling pathway are capable of affecting or even determining cell fate. The depth of their relationship is a captivating topic yet to be explored further. The collision between these two essential pathways could have profound impacts on various pathophysiological processes, not limited to hepatocarcinogenesis. Secondly, what happens after Rictor and p53 are bound? While the most well-known role of Rictor is to facilitate the phosphorylation of Akt on Ser473, it is also a component of an E3 ligase complex, accelerating the ubiquitination and then degradation of c-Myc and cyclin E in colorectal cancer [25]. Whether Rictor modulates p53 through phosphorylation or ubiquitination remains to be determined. Thirdly, we present a stage-specific model of hepatocarcinogenesis based on our observations of Rictor nucleocytoplasmic redistribution. However, it is imperative to note that this model requires further validation. A fundamental question arises: what are the underlying mechanisms and factors driving Rictor's translocation? Investigating alterations in the subcellular location of Rictor offers an intriguing avenue for comprehending its role in cancer progression. All of these interesting hypotheses related to Rictor need more precise investigation before conclusions are drawn.

## Conclusion

In conclusion, our study elucidates the significant role of Rictor, an essential component of mTORC2, in p53^wt^ deactivation in hepatocellular carcinoma (HCC). We reveal an abnormal and relatively tissue-specific interaction between p53 and Rictor during hepatocarcinogenesis, underscoring the importance of cytoplasmic shuttling of Rictor in regulating HCC progression. These findings not only improve our understanding of the mechanisms behind p53 inactivation in HCC (independent of genetic mutations), but also provide a promising avenue for HCC therapy by targeting hard-to-target p53.

### Supplementary Information

**Additional file 1: Supplementary figures**. **Figure S1**. Schematic illustration of animal model generation methods. **Figure S2**. Impact of miR-192 on Rictor mRNA levels and bioinformatic target predictions. **Figure S3**. The effects of miR-192 and Rictor in BxPC3 and HepG2 cells. **Figure S4**. Immunofluorescence staining via confocal microscopy in Hep3B cells. **Figure S5**. MiR-192 expression levels in rat models. **Figure S6**. Analyses of *TP53 *coding sequences in rat HCC samples. **Figure S7**. Analyses of the *TP53 *coding sequences in representative human HCC samples. **Figure S8**. Forest plots of univariate and multivariate analysis of the correlation of Rictor expression with overall survival (OS) among HCC patients.

**Additional file 2: Supplementary tables**. **Table S1**. Oligonucleotides used for cloning Rictor 3’UTR to pMIR-REPORT. **Table S2**. Primer sequences for PCR. **Table S3**. The information about antibodies used in the study. **Table S4**. Summary of the data which were extracted from the total 376* HCC cases for bioinformatic analysis. **Table S5**. Total 114 out of 724 miRNAs which were consistently and positively expressed in all 3 cases of normal primary human hepatocytes (PHHCs) based on our microarray data (GEO accession number, GSE20077). **Table S6**. Clinical characteristics of the HCC patients from TCGA database. **Table S7**. Univariate analysis and multivariate analysis of the correlation of Rictor expression with overall survival (OS) among HCC patients. **Table S8.** Rictor expression associated with clinical pathological characteristics (logistic regression). **Table S9**. The details of GSEA comparing the low and high Rictor expression data sets. **Table S10**. Primers used for Sanger sequencing. **Table S11**. The functional annotation of miR-192 based on reported articles.

**Additional file 3: Supplementary methods**.

## Data Availability

The data and materials used during the current study are available from the corresponding author.

## Abbreviations

HCC: Hepatocellular carcinoma
HBV: Hepatitis B virus
p53^wt^: Wild-type p53
miRNA: MicroRNA
mTORC2: Mammalian target of rapamycin complex 2
BL: Background liver
PHHCs: Normal primary human hepatocytes
CH: Cancerous hepatocytes
co-IP: Coimmunoprecipitation
CM: Confocal microscopy
IB: Immunoblotting
LIHC: Liver HCC
TCGA: The Cancer Genome Atlas
OS: Overall survival
GSEA: Gene Set Enrichment Analysis
KEGG: Kyoto Encyclopedia of Genes and Genomes
NL: Normal liver
LC: Liver cirrhosis
EMT: Epithelial–mesenchymal transition
rAd-p53: Recombinant adenovirus encoding human TP53
