# Supplementary material for: Rictor mediates p53 deactivation to facilitate the malignant transformation of hepatocytes and promote hepatocarcinogenesis
Source: J Transl Med. 2023 Dec 18;21:919. doi: 10.1186/s12967-023-04799-9 (PMC10729423; doi:10.1186/s12967-023-04799-9)
Supplement: Supplementary file 1 — Additional file 1: Supplementary figures. Figure S1. Schematic illustration of animal model generation methods. Figure S2. Impact of miR-192 on Rictor mRNA levels and bioinformatic target predictions. Figure S3. The effects of miR-192 and Rictor in BxPC3 and HepG2 cells. Figure S4. Immunofluorescence staining via confocal microscopy in Hep3B cells. Figure S5. MiR-192 expression levels in rat models. Figure S6. Analyses of TP53 coding sequences in rat HCC samples. Figure S7. Analyses of the TP53 coding sequences in representative human HCC samples. Figure S8. Forest plots of univariate and multivariate analysis of the correlation of Rictor expression with overall survival (OS) among HCC patients. [file 12967_2023_4799_MOESM1_ESM.pdf]

Figure S1

A

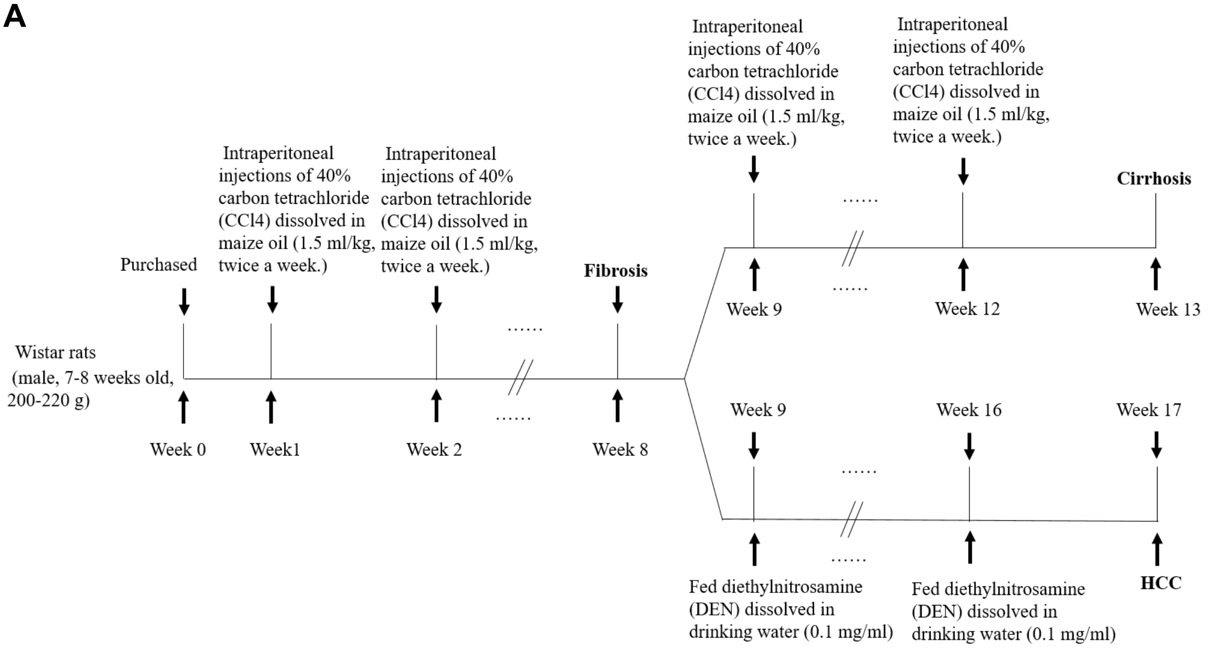

B

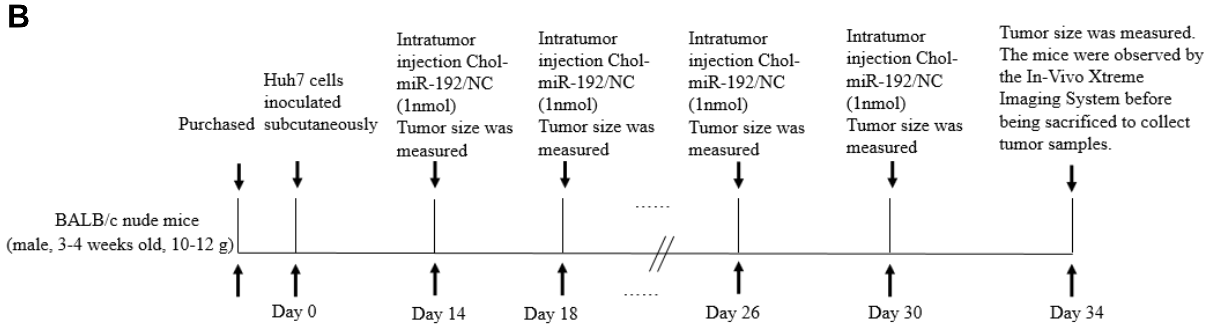

Figure S1. Schematic illustration of animal model generation methods. (A) Establishment of rat models for normal liver, liver fibrosis, liver cirrhosis and hepatocellular carcinoma (HCC). **(B)** Induction of tumor formation in BALB/c nude mice.

Figure S2

A

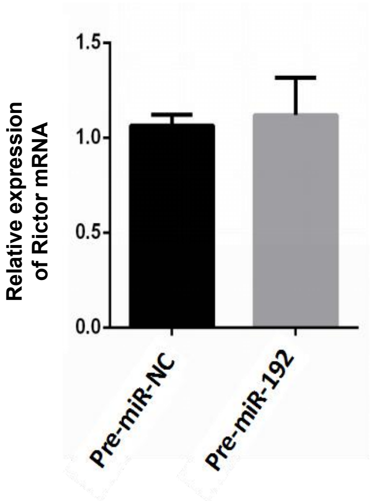

B

Prediction by TargetScan

Conserved

	predicted consequential pairing of target region (top) and miRNA (bottom)	seed match	site-type contri-bution	3' pairing contri-bution	local AU contri-bution	position contri-bution	context score	context score percentile	conserved branch length	P_C
Position 4105-4111 of RICTOR 3' UTR<br>hsa-miR-215	5' ...AGGUUCUGGUGUUUGUAGGUCAC...<br>||||||| 3' CAGACAGUUAAGUAUCCAGUA	7mer-m8	-0.161	0.005	-0.005	-0.013	-0.17	69	2.780	0.2
Position 4105-4111 of RICTOR 3' UTR<br>hsa-miR-192	5' ...AGGUUCUGGUGUUUGUAGGUCAC...<br>||||||| 3' CCGACAGUUAAGUAUCCAGUC	7mer-m8	-0.161	0.020	-0.005	-0.013	-0.15	63	2.780	0.2

Prediction by miRanda

hsa-miR-192/RICTOR Alignment

```
     3' ccGACAGUUAAGUAUCCAGUc 5' hsa-miR-192
          |||  :  :||::|||||||
4094:5' uuCUGGU-GUUUGUAGGUCAc 3' RICTOR
```

mirSVR score: -0.2157
PhastCons score: 0.8170

Figure S2. Impact of miR-192 on Rictor mRNA levels and bioinformatic target predictions. (A) mRNA expression of Rictor was detected by Real-time PCR after transfection with pre-miR-192/pre-miR-NC for 24 hours in Huh7 cells. **(B)** Prediction of the miR-192 binding site within the 3'UTR of Rictor using TargetScan and miRanda.

Figure S3

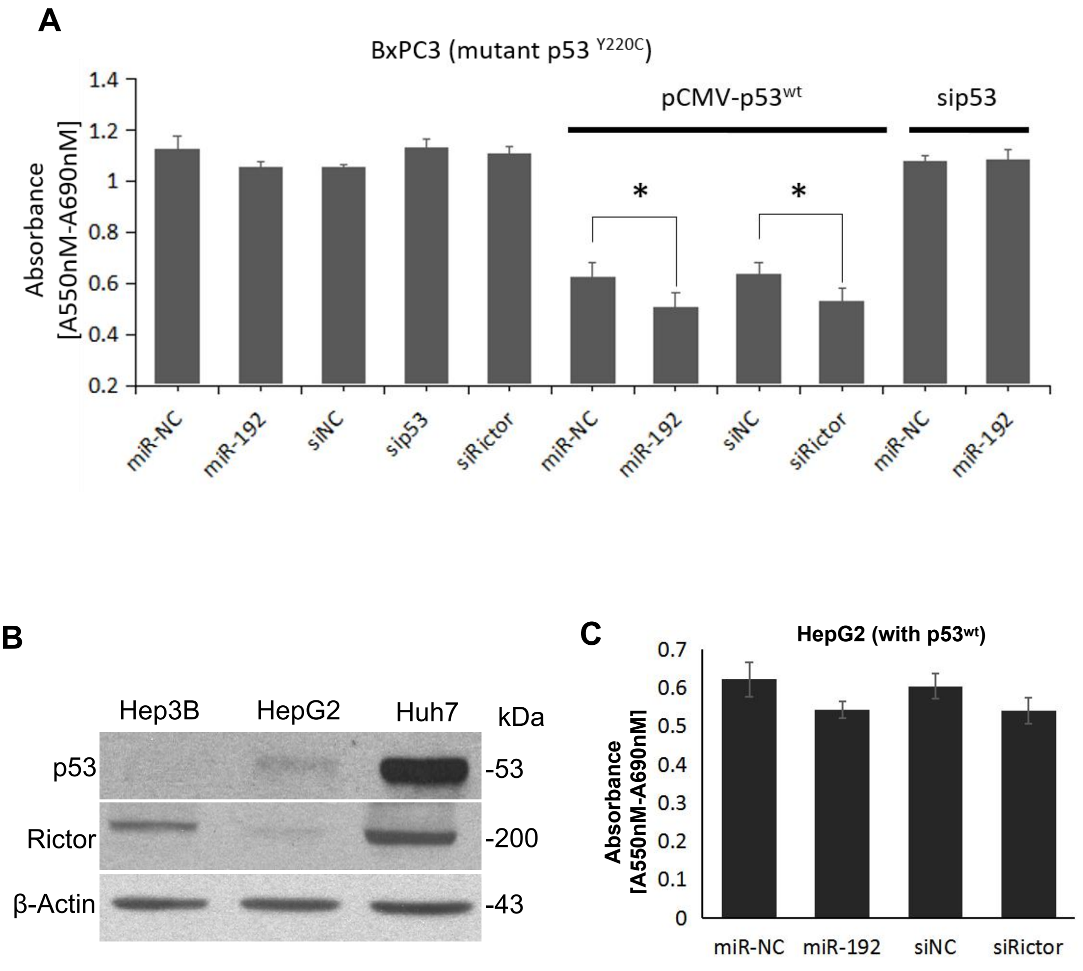

Figure S3. The effects of miR-192 and Rictor in BxPC3 and HepG2 cells. (A) Cell proliferation in pancreatic cancerous cells BxPC3 assessed by MTT assays after 72 hours of transfection with pre-miR-192 or siRictor. **(B)** Western blotting analysis of Rictor and p53 expressions in hepatoma cell lines. **(C)** MTT assays in HepG2 cells (with p53 mutation) transfected with pre-miR-192 or siRictor for 72 hours. MiR-NC and siNC were used as negative controls.

Figure S4

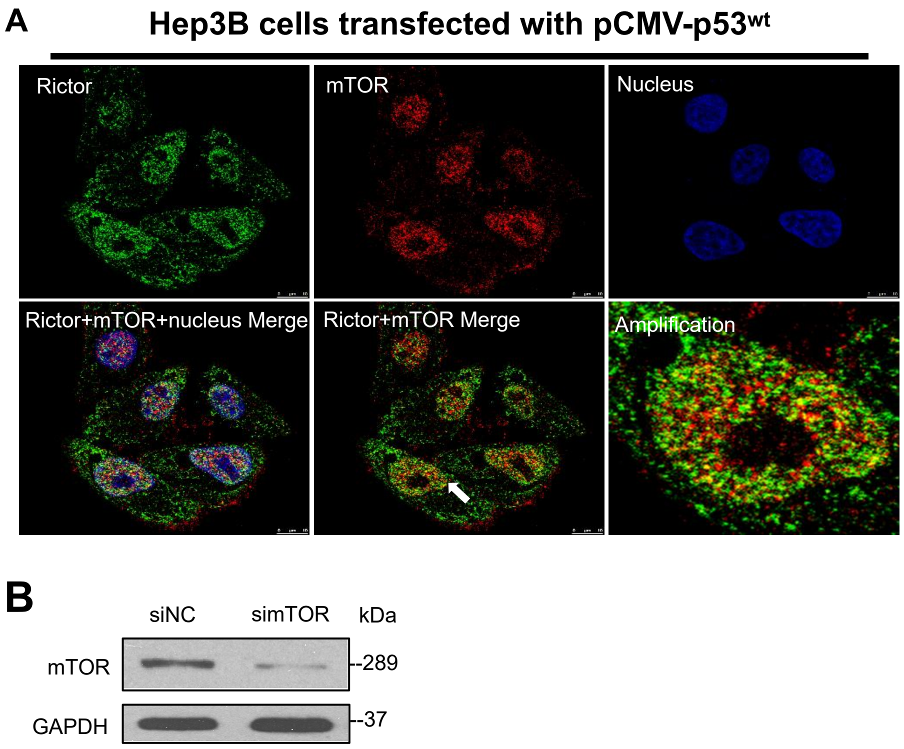

Figure S4. Immunofluorescence staining via confocal microscopy in Hep3B cells. (A) Colocalization of Rictor and mTOR observed through immunofluorescence staining. White arrows indicated the magnified areas. **(B)** mTOR expression assessed by western blotting in Hep3B cells following 48 hours of transfection with simTOR and siNC.

Figure S5

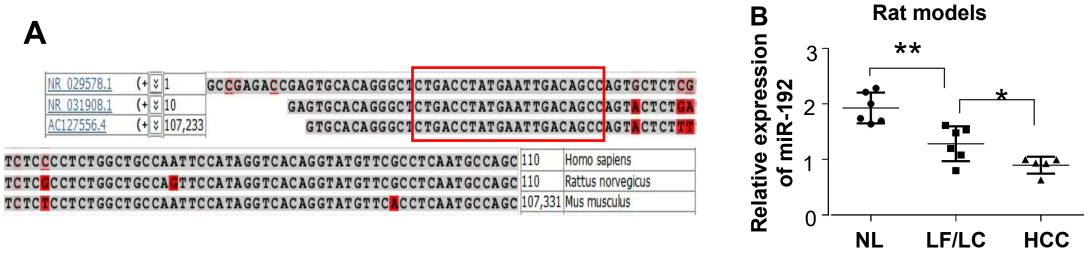

Figure S5. MiR-192 expression levels in rat models. (A) Homology analysis of miR-192 in mice, rats, and humans; the red frame indicates the identical sequence for mature miR-192. **(B)** MiR-192 expression levels measured by qPCR in tissues from rat models, including normal liver (NL, n = 6), liver fibrosis/liver cirrhosis (LF/LC, n = 6), and hepatocellular carcinoma (HCC, n = 5).

Figure S6

A. The example of a mutation site indicated by sequencing analysis

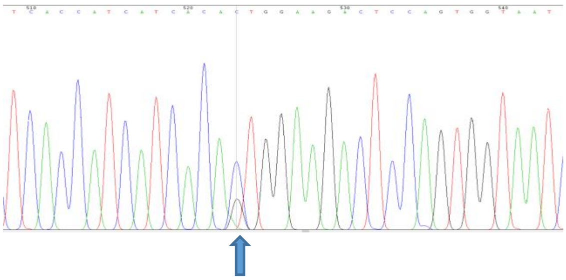

B. HCC1

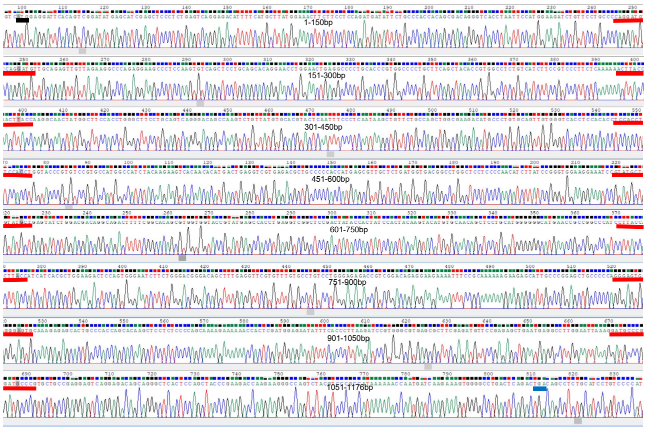

C. HCC2

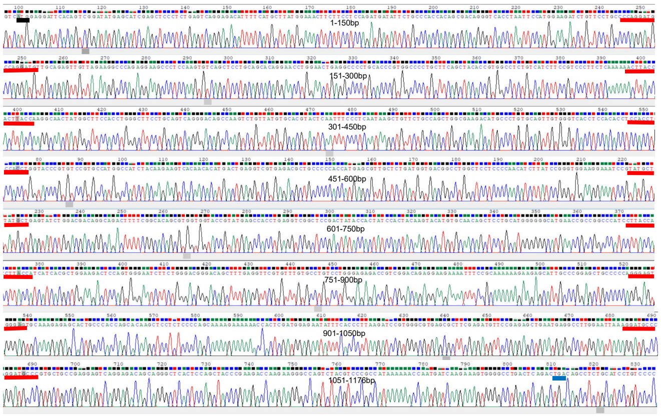

D. HCC3

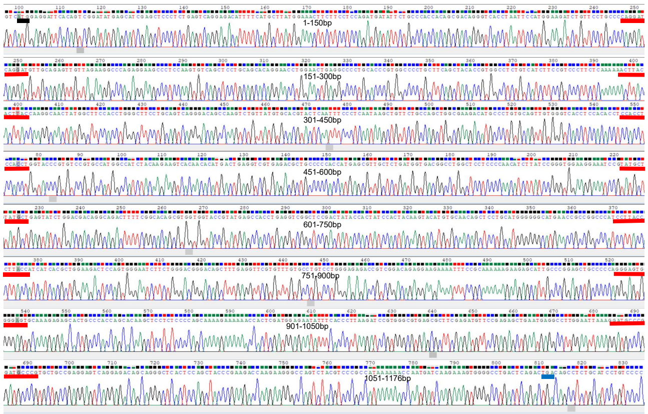

E. HCC4

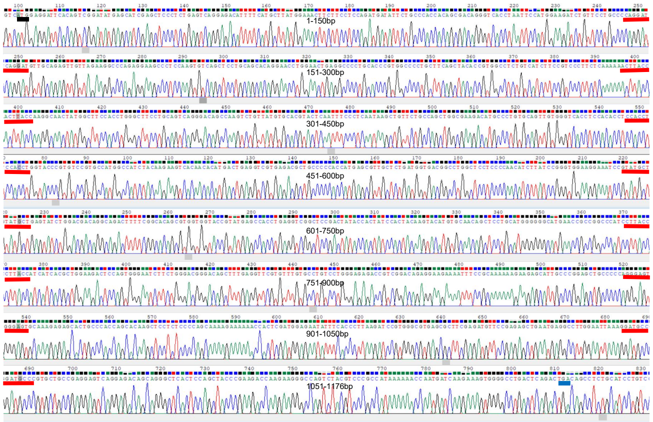

F. HCC5

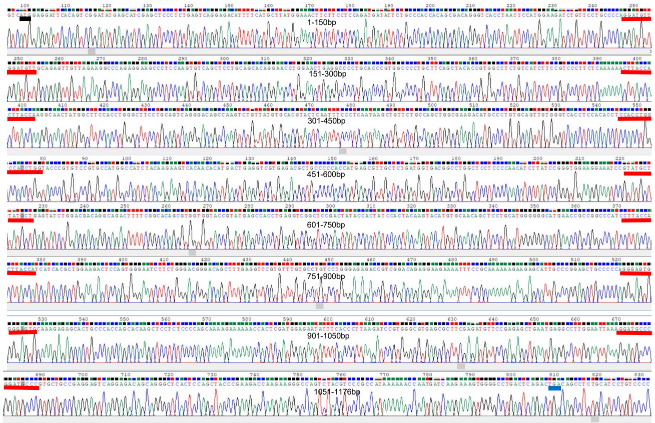

Figure S6. Analyses of *TP53* coding sequences in rat HCC samples. (A) Illustrative example of a mutation site indicated by sequencing analysis. Blue arrow highlighted a double peak observed at the mutation site. **(B-F)** Absence of double across the entire p53 coding sequences, implying the preservation of the wild-type *TP53* gene in all examined rat HCC samples (n=5).

Figure S7

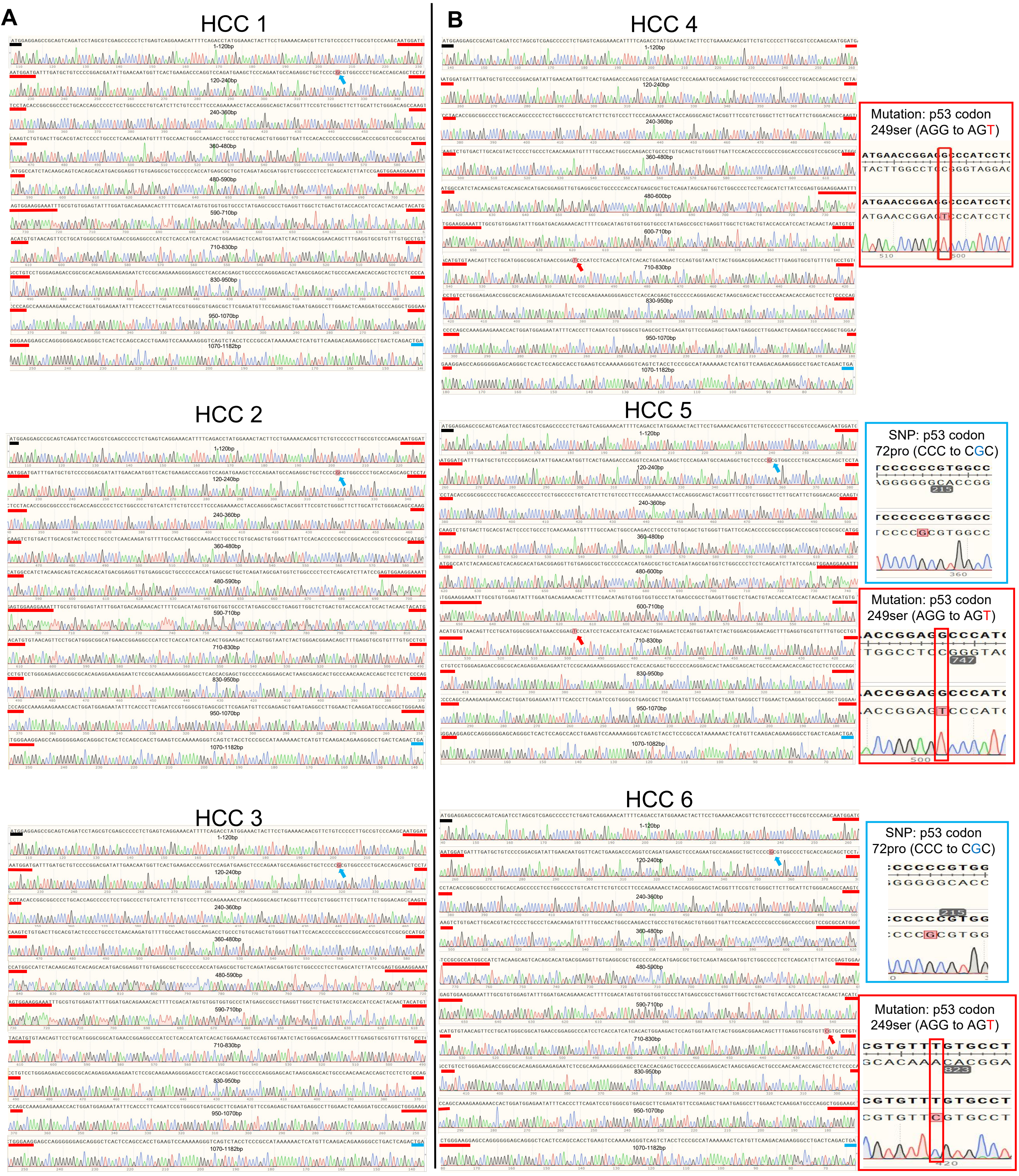

Figure S7. Analyses of the *TP53* coding sequences in representative human HCC samples. (A) The complete p53 coding sequences in three representative HCC samples without *TP53* mutation. **(B)** The complete p53 coding sequences in three representative HCC samples with *TP53* mutation. Comparison of the entire p53 coding sequences and corresponding sites of mutation or single nucleotide polymorphism (SNP) site in representative HCC samples with wild-type (HCC 1–3) and mutant (HCC 4–6) *TP53*. Red arrow indicates the mutation site. Blue arrow indicates the SNP site.

Figure S8

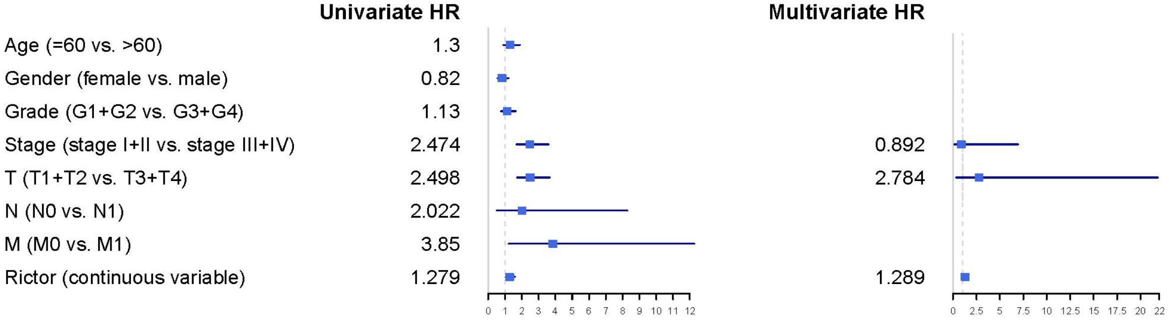

Figure S8. Forest plots of univariate and multivariate analysis of the correlation of Rictor expression with overall survival (OS) among HCC patients. X-axis: Harzard Rtio (HR). The horizontal lines indicate 95% confidence intervals. Vertical dashed lines indicate HR = 1. Y-axis: clinical parameters of the cohort. Each row corresponds to a specific clinical parameter or subgroup within the HCC patient cohort.
