# Supplementary material for: Rictor mediates p53 deactivation to facilitate the malignant transformation of hepatocytes and promote hepatocarcinogenesis
Source: J Transl Med. 2023 Dec 18;21:919. doi: 10.1186/s12967-023-04799-9 (PMC10729423; doi:10.1186/s12967-023-04799-9)
Supplement: Supplementary file 3 — Additional file 3: Supplementary methods. [file 12967_2023_4799_MOESM3_ESM.docx]

**Rictor Mediates p53 Deactivation to Facilitate the Malignant Transformation of Hepatocytes and Promote Hepatocarcinogenesis**

Chun Wang,^1,2†^ Hui Kang,^1,2†^ Yun Yi,^1,2†^ Yang Ding,^1,2^ Fan Wang,^1,2^ Jie Luo,^1,2^ Mingliang Ye,^1,2^ Yinghui Hong,^1,2^ Chao Xia,^3,4^ Junwei Yan,^4,5^ Lan Liu,^1,2^ Jing Liu,^1,2^ Zibiao Zhong,^6^ Zhonglin Zhang,^7^ Qiu Zhao,^1,2*^ Ying Chang,^1,2*^

^1^Department of Gastroenterology, Zhongnan Hospital of Wuhan University, Wuhan, 430071, China;

^2^Hubei Clinical Center and Key Laboratory of Intestinal and Colorectal Diseases, Wuhan, 430071, China;

^3^Department of Geriatrics, Xinhua Hospital, Shanghai Jiao Tong University School of Medicine, Shanghai, 200092, China;

^4^Institute of Liver Diseases, Tongji Hospital, Tongji Medical College, Huazhong University of Science and Technology, Wuhan, 430030, China;

^5^Department of Gastroenterology, Wuhan Central Hospital, Tongji Medical College, Huazhong University of Science and Technology, Wuhan, 430030, China;

^6^Transplant Center of Wuhan University, Institute of Hepatobiliary Diseases of Wuhan University, Zhongnan Hospital of Wuhan University, Wuhan, 430071, China.

^7^Department of Hepatobiliary and Pancreatic Surgery, Zhongnan Hospital of Wuhan University, Wuhan, 430071, China.

| **Contents of the Supplementary data** | **Number** |
| --- | --- |
| **Supplementary methods** | 1 |
| **Supplementary figures** | 8 (Figure S1-S8 was submitted as a pdf file separately) |
| **Supplementary tables** | 11 (Table S1-S11 was submitted as a Excel file separately.) |
| **References** | 2 |

**Supplementary methods**

***Protein preparation and coimmunoprecipitation***

Hep3B cells were transfected with pCMV-p53 for 48 h. Total protein extraction was performed as described previously [1]. Nuclear and plasma proteins were obtained according to the instructions of the protein extraction kit (KGP1100, Jiangsu, China). Resuspend the Magnetic Beads in the vial (tilt and rotate for 2 minutes or gently pipette for 10 times). Transfer 50 ul of Protein A/G Magnetic Beads into a 1.5 mL tube. Add 400 ul of binding/wash buffer to the beads and gently pipette to mix. Place the tube into a magnetic stand to collect the beads against the side of the tube (Hereinafter referred to as magnetic separation). Remove and discard the supernatant. Repeat this step for 2 times. Antibodies (Ab) (anti-p53 (Abcam, Cat#ab26), anti-Rictor (Abcam, Cat#ab70374), anti-mTOR (Abcam, Cat#ab2732)) were diluted by binding/wash buffer to the final concentration of 50 ug/ml. Add 400 ul of diluted antibodies to the Protein A/G Magnetic Beads (Cat#HY-K0202, MedChemExpress LLC, USA). Rotate tube at 4 °C for 2 hours. The Magnetic Beads-Ab complexes were fully suspended by 400 ul of binding/washing buffer. Perform magnetic separation between each wash. Remove and discard the supernatant. Repeat this step for 4 times. Then, 400 ul of the antigen (Ag) were added, fully suspended and incubated in a tumble mixer at 4 °C for 2 hours to allow Ag to bind to the Protein A/G Magnetic Beads-Ab complex. Perform magnetic separation. Remove and discard the supernatant. Wash the Magnetic beads-Ab-Ag complex 4 times using 400 ul binding/wash buffer for each wash. Perform magnetic separation between each wash, remove supernatant and resuspend by gentle pipetting. Add 40 ul SDS-PAGE sample loading buffer (Byotime, China) and mixed well. After boiling denaturation for 5 min, the samples were used for immunoblotting (IB).

***Fluorescence microscopy and confocal microscopy***

Immunofluorescent staining was done as described [2]. Huh7 and Hep3B cells on glass-bottom dishes (NEST, Wuxi, China) were transfected with pCMV-p53 and/or mTOR siRNA (si-mTOR). The cells on glass-bottom dishes were fixed with paraformaldehyde, subjected to immunofluorescence assays and finally imaged under an Olympus DP74 fluorescence microscope or a Leica-LCS-SP8-STED confocal microscope (Leica, Germany).

***Immunohistochemical staining and quantification***

Immunohistochemical staining of patient tissues and animal tissues was performed by Servicebio Company, Wuhan, China, and observed under an inverted microscope (Olympus IX3). Image J (USA) was used to quantified Rictor expression by optical density. For nuclear and cytoplasmic expression pattern analysis, the IHC slides were examined and scored from 0 to 3 multiplied with percentage of stained cells, i.e. H-score (range, 0–300)=0×% of non-stained tumour cells +1×% of weakly stained tumour cells +2×% of moderately stained tumour cells +3×% of strongly stained) based on their expression intensity by two independent pathologists. Five randomly selected high-power (magnification ×400) fields were evaluated in each slide. The ratios of paired nuclear H-score to plasma H-score were calculated.

***Bioinformatic analysis***

All bioinformatics statistical analyses and plots were produced using R (v.3.5.2). The Wilcoxon rank sum test and the Wilcoxon signed rank test were used to analyze gene expression in unpaired and paired samples, respectively. The Wilcoxon rank sum test and logistic regression were used to evaluate relationships between clinicopathological features and gene expression levels. Univariate Cox regression analysis and the Kaplan-Meier method were applied to evaluate prognostic factors. Multivariate Cox regression analysis was used to compare the impact of Rictor expression on overall survival (OS) along with other significant clinical traits.

Gene Set Enrichment Analysis (GSEA) was performed to identify the possible signaling pathways relevant to Rictor and p53. The Kyoto Encyclopedia of Genes and Genomes (KEGG) gene sets (c2.cp.kegg.v6.2.symbols.gmt) from the Molecular Signatures Database (http://www.broad.mit.edu/gsea/msigdb/index.jsp) were used for enrichment analysis. The gene expression data of LIHC patients from the TCGA database were divided into high and low expression phenotypes according to the median expression levels of Rictor and p53. Gene set permutations were performed 1000 times for each analysis. A normal P-value ˂ 0.01 was used to classify the pathways enriched in each phenotype.

KEGG PATHWAY IN CANCER. (https://www.genome.jp/kegg-bin/show_ pathway?hsa05200) includes crucial pathways such as AMPK, PI3K-AKT, JAK-STAT, VEGF, WNT signal pathways that promote proliferation, sustain angiogenesis, or enhance the ability to evade apoptosis and other important cancer phenotypes.

***Genomic DNA extraction and sequencing***

Validation of TP53 mutations in representative rat HCC models and human HCC patients was carried out through Sanger sequencing. Total RNA were extracted from rat paraffin-embedded tissues and fresh HCC tissues, respectively using Trizol (Thermo, USA) and the RNeasy FFPE Kit (#73504, QIAGEN, Germany). The subsequent reverse transcription of mRNA into cDNA followed the manufacturer’s instructions using the Transcriptor First Strand cDNA Synthesis Kit (#04379012001, Roche, Switzerland).

The primer sequences utilized for Sanger sequencing are detailed in Table S10. Library preparation and sequencing services were outsourced to Beijing Tsingke Biotech Co., Ltd. (Beijing, China). All experimental procedures, including RNA extraction, cDNA synthesis, and Sanger sequencing, adhered to the protocols provided with the respective kits.

**References**

1. Chang Y, Yan W, He X, Zhang L, Li C, Huang H, Nace G, Geller DA, Lin J, Tsung A: **miR-375 inhibits autophagy and reduces viability of hepatocellular carcinoma cells under hypoxic conditions.** *Gastroenterology* 2012, **143:**177-187.e178.

2. Du Q, Park KS, Guo Z, He P, Nagashima M, Shao L, Sahai R, Geller DA, Hussain SP: **Regulation of human nitric oxide synthase 2 expression by Wnt beta-catenin signaling.** *Cancer Res* 2006, **66:**7024-7031.
